# Secondhand tobacco smoke exposure and sleep disturbance in school-aged children in Appalachian Ohio

**DOI:** 10.3389/fped.2025.1663801

**Published:** 2025-12-05

**Authors:** Ketrell McWhorter, Christine Kim, Timothy J. Hilbert, Danielle E. McBride, Anthony A. Mangino, Patrick J. Parsons, Kurunthachalam Kannan, Erin N. Haynes

**Affiliations:** 1 Department of Epidemiology and Environmental Health, College of Public Health, University of Kentucky, Lexington, KY, United States; 2 Department of Public Health, School of Health, Calvin University, Grand Rapids, MI, United States; 3 Department of Biostatistics, College of Public Health, University of Kentucky, Lexington, KY, United States; 4 Department of Environmental Health Sciences, College of Integrated Health Sciences, University at Albany, State University of New York, Albany, NY, United States

**Keywords:** tobacco smoke pollution, passive smoking, secondhand smoke, child, sleep, sleep duration, snoring, cotinine

## Abstract

**Introduction:**

Secondhand tobacco smoke (SHS) exposure remains a major public health concern for children and has been implicated in sleep disturbances through biological and behavioral mechanisms. Different methods of measuring SHS exposure have yielded inconsistent results. This study examined the relationship between SHS exposure and sleep among rural Appalachian 7–9-year-olds, utilizing three SHS measurements: natural log-transformed (ln) serum cotinine concentrations, dichotomized serum cotinine levels (≥0.05 vs. <0.05 ng/ml), and parent/caregiver report of ≥1 smoker in the home. We hypothesized SHS-exposed children would have a significantly higher prevalence of sleep disturbance—e.g., short sleep, frequent snoring, night awakenings—compared to unexposed, regardless of measurement method.

**Methods:**

We used data from the Marietta Community Actively Researching Exposure Study, a cohort of 404 children in Marietta and Cambridge, Ohio. Sleep disturbances were parent/caregiver-reported. Linear models were used for continuous outcomes to estimate beta coefficients; log-binomial models with generalized estimating equations (exchangeable correlation) were applied for dichotomized outcomes to estimate prevalence ratios (PR), each with 95% confidence intervals (CI).

**Results:**

Twenty-six percent of children had a parent/caregiver report of ≥1 sleep disturbance. There was a 35% increased prevalence of short sleep [PR = 1.35 (95%CI:1.02–1.80), *p* = .04] with each unit increase of ln serum cotinine levels, adjusting for age, sex, parent education, BMI percentile, and history of breathing difficulties in the past two years. Increasing ln serum cotinine levels were associated with a significant 5.5-min decrease in average sleep duration in age-adjusted models.

**Conclusions:**

Higher SHS exposure was associated with poorer sleep outcomes, with serum cotinine emerging as the only measure linked to short sleep, underscoring the importance of smoke-free environments, particularly in rural communities, and objective exposure assessment.

## Introduction

Although secondhand tobacco smoke (SHS) exposure is declining in the United States (U.S.), it remains a public health concern, particularly among children. The prevalence of SHS among U.S. nonsmokers declined substantially from 87.5% to 25.2% from 1988 to 2014 (1). In 2023, 11% of US adults currently smoked cigarettes, a historic low from its peak prevalence of 42% in 1965 (2). In contrast, SHS exposure remained stable between 2011 and 2012 (25.3%), 2013–2014 (25.2%), and more recently from 2011 to 2018 (25.3%–24.6%) (1, 3). Tobacco smoke is a known human carcinogen also associated with other diseases, such as heart disease (4), and premature death in nonsmoking adults and children (5). In the U.S., an estimated 41,000 deaths among adults each year are caused by SHS (4). Among 3–11, 12–19, and ≥20-year-olds, the 3–11-year-old age group, which aligns with the 7–9-year-old age range represented in the Marietta Communities Actively Researching Exposures Study (CARES) cohort, has the highest percent of nonsmokers with serum cotinine between 0.05 and 0.10 ng/ml (1).

Smoking rates declined faster in urban areas compared with rural areas (6, 7), such as in Central Appalachia America. Additionally, easier access to tobacco products in rural discount stores and higher exposure to SHS in public and private areas make smoking cessation especially difficult in rural vs. urban settings (8). Moreover, Appalachian regions have some of the highest rates of poor sleep in the U.S. According to the 2020 Behavioral Risk Factor Surveillance System, crude and age-adjusted prevalence of short sleep in the highest quintile were counties clustered in Southeastern U.S. and along the Appalachian Mountains (9). According to the National Survey of Children's Health (2020–2021), the prevalence of short sleep among children ages four months to 14 years was highest in Appalachian states, with Ohio reporting a prevalence of 35.9 (95%CI:32.8–39.2) (10).

An increasing number of epidemiologic studies have examined the link between SHS exposure and poor sleep outcomes, including sleep disruption, reduced overall sleep quality, and increased daytime sleepiness (11–13). Among nonsmokers, exposure to SHS results in the inhalation of 60%–80% of airborne nicotine, leading to the absorption of nicotine concentrations similar to those of active smokers (14). Children may be uniquely vulnerable because the proportion of SHS deposited in a child's lungs is greater than in adults due to differences in the diameters and configuration of the airways (15). Nicotine, the primary active compound in tobacco smoke, is a stimulant that promotes arousal and alertness by activating cholinergic neurons in the basal forebrain and disrupting the balance of neurotransmitters involved in sleep regulation (16). These effects can contribute to difficulty falling and staying asleep. SHS exposure, as indicated by elevated cotinine levels in nonsmokers, has been associated with adverse sleep-related outcomes in both children and adults (17–19).

More than 70% of nicotine metabolizes into cotinine, which serves as the best objectively measured biological indicator for assessing SHS exposure and body burden (20, 21). The established range of 0.05–10 ng/ml for serum cotinine has historically been used to define SHS exposure. However, a more recent study analyzed samples using an expanded range of 0.015–10 ng/ml, potentially resulting in higher estimates of the population-level burden of SHS (3). Currently, a level at which serum cotinine has not been associated with adverse health effects has not been identified (1, 22). Even when objective measures are used to explore the relationship between serum cotinine and various sleep outcomes, conflicting results within and among studies have emerged. Some studies have identified significant associations when cotinine is measured continuously (17), while others have observed non-significant findings when using categorical cotinine thresholds (23). Further, higher odds of increased sleep problems among children were found when parent/caregiver report of SHS exposure was used (12). In contrast, findings by Włodarska et al. found more apnea-hypopnea events among children exposed to tobacco smoke when questionnaires were administered to parent/caregivers, but they found no statistically significant differences in waking, snoring, or sleep apnea (24). Variability in SHS-related sleep findings may reflect differences in how exposure is measured. Interviewer-administered questionnaires, adult respondents, and observational study designs yield more accurate SHS assessments, especially when paired with cotinine biomarkers (15).

Given the conflicting methods for assessing SHS exposure and its relationship with sleep in the literature, and the importance of understanding how different assessment approaches relate to reported sleep disturbances in children, this study aimed to investigate the association between SHS exposure using three different methods of SHS assessment and parent/caregiver report of sleep disturbances in a rural Appalachian American pediatric cohort. We hypothesized that SHS exposure, whether measured by (1) ln serum cotinine levels continuously (ng/ml), (2) dichotomously (≥.05 vs. <.05 ng/ml), or by (3) parent/caregiver report of ≥1 smoker in the home, would be associated with shorter sleep duration among children. Secondary analyses explored associations between cotinine and other sleep characteristics, including night awakenings and snoring.

## Methods

CARES consists of a longitudinal cohort of 404 rural Appalachian children living in Marietta and Cambridge, Ohio, and surrounding areas. Children were recruited at ages 7–9 years from 2008 to 2013. Their biological mothers resided in the study catchment area since the 16th week of pregnancy (25). Study design, recruitment, and data collection and compilation methods have been described in detail elsewhere (26–28). All parents/caregivers signed informed consent and all child participants signed informed assent forms approved by the Institutional Review Board of the University of Cincinnati (UC IRB #2011-2542).

This paper focuses on three methods for measuring SHS exposure: continuously measured ln serum cotinine concentrations, dichotomized ln serum cotinine levels (≥0.05 vs. <0.05 ng/ml), and parent/caregiver report of ≥1 smoker in the home. Methods of collecting and analyzing child serum cotinine in the CARES cohort have been described in detail elsewhere (26–29). Briefly, venous whole blood was collected by trained phlebotomists from the antecubital vein into a standard red-top tube (no preservative) and allowed to clot at room temperature for 15–25 min prior to centrifugation to obtain serum off the clot. Serum samples were frozen at a temperature of −20°C until shipped monthly for analysis to the New York State Department of Health's Wadsworth Center, Albany, New York. Serum cotinine analysis was performed using liquid chromatography/tandem mass spectrometry similar to that described by the Centers for Disease Control and Prevention (CDC) for the National Health and Nutrition Examination Survey (*N*HANES) (30) and New York State Wadsworth Laboratories for the New York City Health and Nutrition Examination Survey (31). The method limit of detection (LOD) was 0.05 ng/ml (26). Instrumental values below the LOD were provided by the laboratory and included for statistical analyses; values below 0 were imputed with the LOD divided by the square root of 2 for statistical analyses (32, 33).

Natural log transformation of serum cotinine concentrations aids in normalizing the data distribution and provides a quantitative measure of recent exposure to nicotine, reflecting a higher precision than self-reports. Dichotomizing serum cotinine levels at a threshold of 0.05 ng/ml (i.e., the LOD), although subject to some data loss, serves as a practical approach to classify individuals into exposed vs. non-exposed groups, simplifying the analysis and interpretation of SHS exposure effects on sleep outcomes. Finally, parent/caregiver-reported measures of ≥1 smoker in the home, while subject to reporting bias, nevertheless offer valuable information on chronic SHS exposure in household settings and are vital for assessing the social and behavioral contexts of exposure.

### Assessment of sleep outcomes

A sleep survey was administered to the participant's parent/caregiver during the clinic visit when the participant's blood was collected. Parent/Caregiver-reported sleep outcomes included average sleep duration, snoring frequency, and night awakenings. Average daily sleep duration for each participant was calculated by summing parent/caregiver-reported sleep across four categories—typical weekdays during the school-year (×193), typical weekends during the school-year (×80), typical weekdays during the non-school-year (×68), and typical weekends during the non-school-year (×24)—and dividing the total by 365. While the National Sleep Foundation defines short sleep as <9 h of nightly sleep for this age group (34), we defined short sleep as <8 h and recommended sleep as 8–12 h. The lower 8-h threshold potentially captures true short-sleepers who might otherwise be misclassified as getting sufficient sleep if parents or caregivers report inaccurately. Child snoring loudly was determined by asking the parent/caregiver, “How often does your child snore loudly?” The parent/caregiver could answer “Never,” “Rarely (less than 1 time a week),” “Sometimes (1 to 2 times a week),” “Frequently (3 to 4 times a week),” “Almost always (5 to 7 times a week),” or “Don't know.” Snoring frequency was dichotomized as “<3 times per week” and “≥3 times per week” (frequent) (35)*.* Child night awakenings were determined by asking the parent/caregiver, “After going to sleep at night, on average, how many times will your child awaken before morning?” The parent/caregiver could answer “0,” “1,” “2,” “3,” “4,” “5 or more,” or “Don't know.” Child night awakenings were dichotomized as “<3 times per night” and “≥3 times per night” (frequent) (35). Number of sleep disturbances (i.e., short sleep, frequent snoring, and frequent night awakenings) was dichotomized as “None” or having ≥1 (one, any combination of two, or all three).

### Potential confounders

Key covariates and confounders of interest were identified *a priori* based on existing literature examining the relationship between exposure to SHS and sleep patterns in children (12, 13, 17, 36, 37). Individual factors included child age and sex (12, 13, 17, 36, 37). Additionally, parent education was measured continuously using the Barratt Simplified Measure of Social Status (education subscale), which ranges from 0 to 19 based on the highest level of education attained.^1^ Health status covariates included child sex-adjusted body mass index (BMI) percentile (36) and parent/caregiver report of repeated episodes of difficulty breathing (13, 17) in the past 2 years. BMI categories were calculated using the R package “growthcleanr” (38) and BMI percentile based on the CDC growth charts at the 5th, 85th, and 95th percentile by age. Underweight: <5th percentile; Normal: 5th to <85th percentile; Overweight: 85th to <95th percentile; Obese: ≥95th percentile). Repeated episodes of breathing difficulty in the past 2 years were determined by asking the parent/caregiver, “During the last two years, has your child had repeated episodes of any of the following health conditions?” The parent/caregiver could answer “Yes,” “No,” or “Don't know” to the following conditions: “asthma,” “cough,” “trouble breathing,” “chest tightness,” “bronchitis,” or “none of the above.”

### Statistical analysis

Demographic characteristics were summarized using means and standard deviations (SD) for continuous variables and counts and proportions for categorical variables. Serum cotinine was transformed using the natural logarithm (ln) due to skewed data distributions. Due to the right-skewed distribution of serum cotinine, geometric means (GM) were used instead of arithmetic means, as they are less influenced by extreme values and more appropriate for skewed data. Additionally, GMs align with the back-transformation of ln-transformed serum cotinine values, making interpretation and reporting more coherent. To improve interpretability, cotinine effects were expressed per doubling of serum cotinine concentration (log₂ scale) in Supplementary Tables 1–3. We used generalized estimating equations (GEE) with an exchangeable working correlation structure approach within the generalized linear model (GLM) framework to analyze all outcomes. A linear model was specified for the continuous outcome (average sleep duration), utilizing a Gaussian distribution, and a log-binomial model was employed for the dichotomous sleep outcomes, using a binomial distribution with a log link function. Using GEE allowed robust estimation of prevalence ratios (PRs), while still accounting for potential correlation among children from the same household without estimating random variance. Where log-binomial models did not converge due to the sparseness of data, we applied a Poisson regression model with robust variance estimation as an alternative method to obtain valid PR estimates for binary outcomes. To assess the strength and consistency of our primary log-binomial findings across modeling strategies, we also fit generalized linear mixed models (GLMMs using PROC GLIMMIX in SAS) with a Laplace likelihood approximation under a fixed-effects specification (no random intercept). Given multiple exposure definitions and sleep outcomes, we recognize the potential for a Type I error. Because this study was exploratory, no formal correction for multiple testing was applied; instead, consistency of direction and magnitude across outcomes was emphasized. Age-adjusted models were assessed in addition to fully adjusted models, which accounted for age, child sex, parent education, BMI percentile, and history of breathing difficulty in the past 2 years*.* All significance tests were two-sided, and a *p*-value of <0.05 was considered statistically significant. Statistical analyses were completed using R (39).

## Results

### Study population characteristics

Table 1 shows sociodemographic, clinical, and parent/caregiver characteristics, lifestyle behaviors, and serum cotinine levels among 404 children in the Marietta CARES cohort. The mean age of the participants was 8.4 years ± standard deviation (SD) of 0.9, and 46% were female. Five percent of children were non-white, and 24% lived in a single-parent household. Overall, the average Barratt score for parent education was 14.8 ± 2.5. Approximately 33% of children had a BMI ≥85th percentile (overweight or obese). Nearly 40% of children experienced repeated episodes of asthma, cough, trouble breathing, chest tightness, or bronchitis within the last 2 years, and 37% of all children lived in households with ≥1 smoker in the home. The average daily number of cigarettes smoked per household was 20.3 ± 13.1. The geometric mean for serum cotinine was 0.08 ± 7.8 ng/ml, and 50% of children had serum cotinine levels ≥0.05 ng/ml. Across increasing levels of household smoking (0, 1, and 2–3 smokers), the geometric mean serum cotinine concentrations were 0.03, 0.37, and 1.19 ng/ml, respectively. Average sleep duration for the cohort was 9.3 ± 1.1 h per night; 6% of children had parent/caregiver-reported short sleep; 20.3% of participants had parent/caregiver-reported frequent snoring; 4.2% had frequent night awakenings, and 26.2% of children had parent/caregiver report of ≥1 sleep disturbance.

**Table 1:** Demographic characteristics of Marietta CARES pediatric cohort (7–9 years old) by sleep index, 2008–2013, all and by number of poor sleep behaviors reported, *n*=404.

Child Characteristics	All
	*N*=404
Sociodemographic Characteristics	Mean (SD) or *n* (%)
Age (years), *n* = 404	8.4 (0.9)
Sex, *n* = 404	
Female	186 (46.0%)
Child Ethnicity, *n* = 404	
White	384 (95.1%)
Non-white	20 (5.0%)
Parents living in household, *n* = 392	
1	97 (24.7%)
2	295 (75.3%)
Parent Education Score (Barratt), *n* = 402	14.8 (2.5)
Clinical Characteristics	
BMI-for-Age Percentile, *n* = 404	63.1 (30.3)
BMI-for-Age Class, *n* = 404	
<85th percentile	269 (66.6%)
85th to <95th percentile	61 (15.1%)
≥95th percentile	74 (18.3%)
Repeated episodes of breathing difficulty in last 2 years, *n* = 402	
Asthma	53 (13.2%)
Cough	120 (29.9%)
Trouble breathing	47 (11.7%)
Chest tightness	28 (7.0%)
Bronchitis	54 (13.4%)
Any (at least one)	157 (39.1%)
None	247 (61.4%)
Sleep Behaviors	
Total Average Sleeping (hours), *n* = 403	
Mean	9.3 (1.1)
<8 h/night	24 (6.0%)
≥8 h/night (recommended)	379 (94.0%)
Child snore, *n* = 400	
≥3 times/week	81 (20.3%)
<3 times/week (recommended)	319 (79.8%)
Child wakes, *n* = 402	
≥3 times/week	17 (4.2%)
<3 times/week (recommended)	385 (95.8%)
Number of poor sleep behaviors reported	
≥1	106 (26.2%)
None	298 (73.8%)
Internal Dose Markers of Exposure	
Serum Cotinine (ng/ml), *n* = 329	
Geometric mean (SD)	0.08 (7.8)
≥0.05 ng/ml	163 (49.5%)
<0.05 ng/ml	166 (50.4%)
Parent/Caregiver Characteristics	
Household smoking information, *n* = 404	
Smoker in the HH, yes	148 (36.6%)
Number of smokers in the HH	
1	101 (68.2%)
2–3	47 (31.8%)
Daily cigarettes smoked in HH	20.3 (13.1)

SD, standard deviation; HH, household.

### SHS exposure and parent/caregiver-reported sleep disturbance

#### Continuously measured ln serum cotinine concentrations

After adjusting for age, increasing levels of ln serum cotinine were significantly associated with prevalence of short sleep [PR = 1.37 (95% CI: 1.10, 1.71), *p* = .005], prevalence of frequent snoring [PR = 1.13 (95% CI: 1.02, 1.26), *p* = 0.03], and prevalence of having ≥1 sleep disturbance [PR = 1.14 (95% CI: 1.04, 1.25), *p* = .007] (Table 2). Similarly, in age-adjusted models, a one-unit increase in log₂ cotinine (a doubling of serum cotinine concentration) was associated with a 24.0% higher prevalence of short sleep (PR = 1.24, 95% CI: 1.07–1.45, *p* *=* *0.005*), 9.0% higher prevalence of frequent snoring (PR = 1.09, 95% CI: 1.01–1.17, *p* *=* *0.03*), and having ≥1 sleep disturbance (PR = 1.09, 95% CI: 1.02–1.17, *p* *=* *0.007*) (Supplementary Table 1). After further adjusting for child sex, parent education, BMI percentile, and history of breathing difficulty, increasing levels of ln serum cotinine remained significantly associated with short sleep [PR = 1.35, (95% CI: 1.02, 1.80), *p* = .04] (Table 2) and a doubling of serum cotinine concentration was associated with an 11.0% increased prevalence of short sleep (PR = 1.11, 95% CI: 1.05–1.51, *p* *=* *0.04*) (Supplementary Table 1). Having ≥1 sleep disturbance [PR = 1.11 (95% CI: 0.99, 1.23), *p* = .07] approached significance but was not significant in the fully adjusted models.

**Table 2:** Prevalence ratios (PR) and 95% confidence intervals (CI) for parent/caregiver-reported sleep disturbances by three measures of SHS exposure (continuously measured ln serum cotinine, dichotomized cotinine ≥.05 vs. <.05 ng/ml, and parent/caregiver report of ≥1 smoker in the household vs. none) in the Marietta CARES pediatric cohort (7–9 years old), 2008–2013, *n* = 404.

Continuously measured Natural Log-Serum Cotinine						
Sleep Behavior	Age-adjusted Association between Natural Log-Serum Cotinine and Sleep Behaviors			Fully Adjusted Association between Natural Log-Serum Cotinine and Sleep Behaviors		
	PR	95% CI	*p*-value	PR	95% CI	*p*-value
Average Sleep (<8 vs. ≥8 h)	**1.37**	**1.10–1.71**	**0.005**	**1.35**	**1.02–1.80**	**0.04**
Snoring (≥3 vs. <3 x/week)	**1.13**	**1.02–1.26**	**0.03**	1.10	0.98–1.25	0.11
Night Awakenings (≥2 vs. <2 x/night)	1.28	0.96–1.70	0.10	1.23	0.81–1.87	0.34
Number of sleep disturbances reported (≥1 vs. none)	**1.14**	**1.04–1.25**	**0.007**	1.11	0.99–1.23	0.07
Dichotomized (≥0.05 vs. <0.05 ng/ml)						
Sleep Behavior	Age-adjusted Association between Dichotomized Natural Log-Serum Cotinine and Sleep Behaviors			Fully Adjusted Association between Dichotomized Natural Log-Serum Cotinine and Sleep Behaviors		
	PR	95% CI	*p*-value	PR	95% CI	*p*-value
Average Sleep (<8 vs. ≥8 h)	**3.62**	**1.22–10.7**	**0.02**	2.89	0.77–10.9	0.12
Snoring (≥3 vs. <3 x/week)	1.51	0.96–2.37	0.08	1.32	0.81–2.13	0.27
Night Awakenings (≥2 vs. <2 x/night)	2.57	0.65–10.2	0.18	1.84	0.51–6.66	0.35
Number of sleep disturbances reported (≥1 vs. none)	1.45	0.99–2.13	0.06	1.21	0.79–1.85	0.37
Parent/Caregiver-report of ≥1 Smoker in the Home						
Sleep Behavior	Age-adjusted Association between Parent/Caregiver Report of ≥1 Smoker in the Home and Sleep Behaviors			Fully Adjusted Association between Parent/Caregiver Report of ≥1 Smoker in the Home and Sleep Behaviors		
	PR	95% CI	*p*-value	PR	95% CI	*p*-value
Average Sleep (<8 vs. ≥8 h)	**2.40**	**1.07–5.38**	**0.03**	1.88	0.68–5.19	0.22
Snoring (≥3 vs. <3 x/week)	1.43	0.95–2.13	0.08	1.21	0.79–1.85	0.38
Night Awakenings (≥2 vs. <2 x/night)	2.64	0.97–7.20	0.06	2.13	0.65–7.05	0.21
Number of sleep disturbances reported (≥1 vs. none)	1.39	0.98–1.95	0.06	1.16	0.80–1.68	0.42

Age-adjusted model includes age (years).

The fully adjusted model additionally considers the following covariates: child sex, parent education, BMI percentile, and history of breathing difficulty in the past 2 years.

Bold font indicates significant associations (*p* < .05).

#### Dichotomized (≥.05 vs. <.05 ng/ml) ln serum cotinine concentrations

In age-adjusted models, children with serum cotinine levels ≥0.05 ng/ml (i.e., detectable) were over 3.5 times more likely to have short sleep duration compared to children with non-detectable levels, i.e., <0.05 ng/ml [PR = 3.62, (95% CI:1.22, 10.7), *p* = .02] (Table 2). However, this relationship became weaker in fully adjusted models.

#### Parent/caregiver report of ≥1 smoker in the home

In age-adjusted models, children with parent/caregiver report of having ≥1 smoker in the home were nearly 2.5 times more likely to have short sleep duration compared to children with no smokers in the home [PR = 2.40, (95% CI: 1.07, 5.38), *p* = .03] (Table 2). This relationship fell below the threshold for significance in fully adjusted models. Frequent night awakenings [PR = 2.64, (95% CI: 0.97, 7.20), *p* = .06] and having ≥1 sleep disturbance [PR = 1.39, (95% CI:0.98, 1.95), *p* = .06] reached near significance in age-adjusted models and further attenuated in fully adjusted models (Table 2). Fully adjusted log-binomial models failed to converge in 3 instances: when ln serum cotinine was included as a dichotomous predictor of (1) average sleep duration and (2) night awakenings, and (3) when having ≥1 smoker in the home was modeled as a predictor of average sleep duration (Table 2). In these cases, Poisson regression with robust variance estimation was used as an alternative to obtain interpretable PR estimates and showed no relationship. All other models converged successfully. During robustness checks, we observed mostly no changes in parameter estimates and direction when using PROC GLIMMIX with a Laplace likelihood approximation model, with a few very minor exceptions of significance. Compared to borderline significance in GEE, the following relationships became significant: (1) between increasing ln serum cotinine levels and frequent night awakenings (PR = 1.28, 95% CI: 1.01–1.63, *p* = 0.04) in the age-adjusted model, (2) increasing ln serum cotinine levels and having ≥1 sleep disturbance (PR = 1.11, 95% CI: 1.00–1.24, *p* = 0.048) in the fully adjusted model, and serum cotinine levels ≥0.05 ng/ml and having ≥1 sleep disturbance (PR = 1.46, 95% CI: 1.00–2.12, *p* = 0.049) in the age-adjusted model. We observed the same patterns when using log₂-transformed (per doubling) serum cotinine (Supplementary Table 3). These results confirm that log-binomial findings were not sensitive to modeling approach.

#### Continuously measured ln serum cotinine concentrations and average sleep duration

In age-adjusted models, the coefficient for ln serum cotinine was *β*=−0.09, 95% CI: −0.15, −0.03, *p* = .0003, suggesting a 5.49-min decrease in average sleep duration with increasing ln serum cotinine (Table 3). However, in fully adjusted models, significance was lost. Only parent education had a significantly positive association with average sleep duration [*β* = 0.06, (95% CI: 0.01, 0.11), *p* = .01], independent of other model covariates, corresponding to an approximate 4-min increase in sleep duration. We observed the same patterns in estimate and direction when using log₂-transformed (per doubling) serum cotinine (Supplementary Table 2).

**Table 3:** Regression coefficients and 95% confidence intervals (CI) from generalized estimating equation models for average sleep duration and ln serum cotinine levels in Marietta CARES pediatric cohort (7–9 years old), 2008–2013, *n* = 404.

Age-adjusted	*n* = 404	Average sleeping hours∼ln serum cotinine		
		Estimate (95% CI)	*p*-value	in minutes
(Intercept)		9.67 (8.66–10.7)	**<.0001**	
ln cotinine		**−0.09 (−0.15, −0.03)**	**0.003**	**−5.49**
Age (years)		**−**0.07 (−0.18, 0.05)	0.25	**−**3.94
Fully Adjusted	***n* = 404**	**Estimate (95% CI)**	***p*-value**	**in minutes**
(Intercept)		8.92 (7.73, 10.1)	**<.0001**	
Ln cotinine		**−**0.06 (−0.13, 0.01)	0.12	**−**3.42
Age (years)		**−**0.06 (−0.17, −0.05)	0.31	**−**3.44
Sex		0.03 (−0.17, 0.24)	0.73	2.11
Parent education		**0.06 (0.01, 0.11)**	**0.01**	**3.61**
BMI percentile		0.001 (−0.004, 0.002)	0.61	**−**0.05
Episode		**−**0.02 (−0.24, 0.20)	0.84	**−**1.33

Parent education: Barratt simplified measure of social status (education subscale).

Episodes: repeated episodes of difficulty breathing in the past 2 years.

The fully adjusted model additionally considers the following covariates: age, child sex, parent education, BMI percentile, and history of breathing difficulty in the past 2 years.

Bold font indicates significant associations (*p* < .05).

## Discussion

Twenty-six percent of children had a parent/caregiver report of ≥1 sleep disturbance. In fully adjusted models using serum cotinine as a continuous measure, prevalence of short sleep duration (35%) attenuated but remained significant. Frequent snoring was not significant, with having ≥1 sleep disturbance (11%) reaching borderline significance. In age-adjusted models, children were 3.6 times more likely to have short sleep duration with serum cotinine levels ≥.05 ng/ml (vs. <.05 ng/ml) and nearly 2.5 times more likely to have short sleep duration with ≥1 smoker (vs. none) living in the home. Increasing ln serum cotinine levels were associated with a significant 5.5-min decrease in average sleep duration in age-adjusted models. Parent education had a significantly positive association with average sleep duration, independent of other model covariates.

Although our significant associations between SHS exposure and short sleep duration when using continuously or dichotomously measured ln serum cotinine levels were only observed in age-adjusted models, and attenuated in fully adjusted models, previous studies have found associations with increasing serum cotinine and risk of trouble sleeping in fully adjusted models. Yolton et al. found a higher odds ratio of sleep onset delay, parasomnias, sleep-disordered breathing (SDB), daytime sleepiness, and overall sleep disturbance with increasing serum cotinine among children 6–12-years, even after adjusting for covariates such as prenatal tobacco exposure, maternal depression, and household density (17). Likewise, Du et al. found significant correlations between high serum cotinine levels (.05–3.00 ng/ml) and shorter sleep duration among adolescents after fully adjusting for covariates such as poverty income ratio, physical activity, and caffeine intake (18). The contrast in findings may reflect differences in covariate selection across studies. While our models included age, sex, parent education, BMI, and history of breathing difficulty, variations in specific confounders and outcome assessment may influence the observed associations. In contrast to our non-significant results when using parent/caregiver reports of having ≥1 smoker in the home, a review and meta-analysis by Safa et al. found a pooled OR of 1.20 (95% CI: 1.09–1.33; *p* = .0003; *n* = 692,690 adolescents and adults) for short sleep duration among the SHS-exposed vs. not exposed groups after adjusting for covariates (19). Similar to our study, all studies in the meta-analysis assessed SHS exposure using self-reported questionnaires and interviews. It should be noted, as stated by the authors, that there was large heterogeneity between the studies (*I*^2^ = 68%, *p* = .004), indicating variation in effect sizes across 7 studies that examined short sleep duration, and only 2 of the 7 studies included adolescents (19). Similarly, Reichenberger et al. found that SHS exposure was associated with reduced sleep duration at age 5, with an even greater reduction by age 9 in longitudinal analyses. Cross-sectional analyses at age 9 also showed a negative association between SHS exposure and sleep duration when assessed through caregiver interviews (13). In contrast, Davila et al. found no significant associations between SHS-exposed nonsmokers and reports of sleep disorders or symptoms [OR = 1.43 (95% CI: 0.79–2.57)] (22); however, their study was conducted in an adult population (mean age: 48.3 ± 0.29 years, *n* = 4,123). While our study aligned with several key conditions associated with higher sensitivity and specificity of SHS exposure assessment, including the use of interviewer-administered questionnaires in an observational context and responses provided by adults (15), we did not include repeat questionnaires or cotinine measurements. The absence of repeated measures may have contributed to the non-significant associations observed in our study, despite following other best practices in self-reported exposure assessment. We observed a significantly higher prevalence of children with ≥1 sleep disturbance when using continuously measured ln serum cotinine levels in age-adjusted models, nearly reaching significance after adjusting for all covariates. However, when assessing whether serum cotinine levels ≥0.05 ng/ml were associated with having ≥1 sleep disturbance, no significant associations were found in either age-adjusted or adjusted models. The inconsistencies in our findings, both within this study and compared to previous literature, highlight how results vary depending on the method used to measure SHS exposure.

Our study observed a significantly increased prevalence of frequent snoring among children with increasing ln serum cotinine levels in age-adjusted models; however, this relationship did not reach significance in fully adjusted models. Similar to our non-significant association between SHS exposure and snoring, regardless of the SHS measurement method used, a systematic review of the literature on SHS and SDB in children, characterized by habitual snoring [loud snoring ≥3 nights per week (40)], identified three studies that found no association (41). More notably, however, the review also reported 15 case-control studies that demonstrated a significant association between SHS exposure and SDB.

Associations between SHS exposure (regardless of measurement method) and frequent night awakenings were not statistically significant in either age-adjusted or fully adjusted models. Our findings diverge from the results of a recent cross-sectional study of more than 45,000 children in China, which reported that SHS exposure *in utero* and during the first two years of life was associated with increased odds of sleep disturbances, such as shorter sleep duration, night awakenings, snoring, and breathing problems, at ages 6–18 years (12). The significantly larger sample size in that study may have provided greater statistical power to detect associations that were not evident in our more modestly sized cohort.

There are limitations to our study. Our sample consisted of a relatively small proportion of children with frequent night awakenings (*N* = 17), which may contribute to our lack of significant associations. Additionally, other factors known to be associated with sleep were not included in the dataset: melatonin or other sleep aid use, caffeinated drink consumption, exposure to blue light and/or use of electronic devices in bed, neighborhood noise, and nighttime light exposure. Additionally, parent-reported child sleep measures may be subject to recall bias and may not accurately reflect objective sleep, as the study lacked daily sleep logs and electronic sleep monitoring. Bedtime is an important determinant of sleep duration for children (42). Parent/caregiver-reported sleep duration was assessed at a single time point for typical weekdays and weekends during both the school year and non-school year, limiting the ability to capture sleep patterns or changes over time. As noted above, we also did not measure screen time in bed, which is a well-documented factor associated with delayed sleep onset and reduced sleep duration in children and adolescents (43). Finally, since no formal *p*-value adjustment was applied (e.g., Bonferroni correction or false discovery rate), given the exploratory nature, findings should cautiously be interpreted as hypothesis-generating given multiple tests across related sleep outcomes.

Despite these limitations, this study has numerous strengths. One strength of this study is that the CARES cohort reflects the demographic composition of the Appalachian population. Given that Appalachia's population was 26.6 million in 2023, with non-White people comprising only 21.3% (national rate = 41.6%) and White people comprising 78.7% (national rate = 58.4%), our cohort is comparable to Appalachian communities (44). Moreover, smoking behaviors reported in our cohort align with regional trends: according to the CDC and the Appalachian Regional Commission, adult smoking prevalence in Central Appalachia was 25.2% in 2020 (6), compared to a national rate of 15.2% (45). In our sample, 36.6% of children lived in households with ≥1 smoker, reflecting the elevated household and community-level tobacco burden consistently documented across Appalachian populations (6). Another strength is the use of validated sleep questions and serum cotinine as an objective SHS biomarker, both collected during the same study visit. It should be noted, however, that although serum cotinine has a high specificity and sensitivity with a relatively prolonged half-life of 16–20 h in children (46–48), it provides a relatively short-term snapshot of recent exposure and does not reflect chronic or cumulative exposure (46, 47). Lastly, we utilized three methods for measuring SHS exposure to investigate its association with parent/caregiver-reported sleep disturbances in a rural Appalachian pediatric cohort. Although there have been several previous studies that have found significantly positive relationships between SHS as measured by parent/caregiver-reported tobacco use and sleep disruption among children and adolescents (11–13, 36, 37, 41), yet few have included serum cotinine as an objective internal measure of environmental tobacco smoke exposure in pediatric populations. Geometric mean serum cotinine levels rose with more household smokers; however, parent-reported smoking may underestimate SHS exposure, as 37% reported a smoker while 50% of children had detectable cotinine, indicating possible underreporting or unrecognized sources (27).

## Conclusion

To our knowledge, this is the first study to report on the impact of SHS exposure on sleep disturbances using multiple SHS measurement methods in Appalachian children. Ln serum cotinine as a continuous measure was the only SHS measure associated with a sleep disturbance: a higher prevalence of short sleep duration after adjusting for covariates. Although causal inference cannot be established from this observational design, the consistency of findings across analytic approaches supports a plausible association between tobacco smoke exposure and children's sleep disruption. These findings highlight the need to maintain smoke-free environments where children live, play, and sleep. Parents and caregivers should avoid smoking indoors or in vehicles, and educators should promote awareness of secondhand smoke's impact on sleep and overall health. Community efforts to establish smoke-free homes, schools, and recreational spaces remain essential. Moreover, our findings underscore the importance of using a continuous measure of serum cotinine as an objective measure of SHS exposure when assessing sleep disturbance in child cohorts.

## Data Availability

The data analyzed in this study is subject to the following licenses/restrictions: the dataset is not publicly available, but data can be requested by contacting the PI, erin.haynes@uky.edu. Requests to access these datasets should be directed to erin.haynes@uky.edu.

## Footnotes

1 Barratt W. The Barratt Simplified Measure of Social Status (BSMSS) Measuring SES. Indiana State University (2006). Available online at: http://wbarratt.indstate.edu/socialclass/Barratt_Simplifed_Measure_of_Social_Status.pdf
