# Supplementary material for: Secondhand tobacco smoke exposure and sleep disturbance in school-aged children in Appalachian Ohio
Source: Front Pediatr. 2025 Dec 5;13:1663801. doi: 10.3389/fped.2025.1663801 (PMC12714884; doi:10.3389/fped.2025.1663801)
Supplement: Supplementary file 1 [file Table1.docx]

| **Supplemental Table 1. Prevalence Ratios (PR) and 95% Confidence Intervals (CI) for Parent/Caregiver-Reported sleep disturbances associated with a doubling of serum cotinine concentration (log₂‑transformed) among children in the Marietta CARES pediatric cohort (ages 7–9 years), 2008–2013 (n = 404).** | | | | | | | |
| --- | --- | --- | --- | --- | --- | --- | --- |
| **Sleep Behavior** | Age-adjusted Association between Natural Log-Serum Cotinine and Sleep Behaviors | | |  | Fully Adjusted Model of Association between Natural Log-Serum Cotinine and Sleep Behaviors | | |
|  |  |  |  |  |  |  |  |
|  | **PR** | **95% CI** | **p** |  | **PR** | **95% CI** | **p-value** |
| Average Sleep (<8 hours vs. ≥8 hours) | **1.24** | **1.07-1.45** | **0.005** |  | **1.11** | **1.01-1.51** | **0.04** |
| Snoring (≥3 x/week vs. <3 x/week) | **1.09** | **1.01-1.17** | **0.026** |  | 1.07 | 0.98-1.17 | 0.11 |
| Night Awakenings (≥2 x/night vs. <2 x/night) | 1.18 | 0.97-1.44 | 0.10 |  | 1.16 | 0.85-1.57 | 0.34 |
| Number of sleep issues reported (≥1 vs. None) | **1.09** | **1.02-1.17** | **0.007** |  | 1.07 | 0.99-1.16 | 0.07 |
| Cotinine modeled on log₂ scale. PRs reflect the effect per doubling of serum cotinine concentration. | | | | | | | |
| Age-adjusted model includes age (years). | | | | | | | |
| The fully adjusted model additionally considers the following covariates: child sex, parent education, BMI percentile, and history of breathing difficulty in the past 2 years. | | | | | | | |

| **Supplemental Table 2. Regression Coefficients and 95% Confidence Intervals (CI) from PROC GLIMMIX Fixed-Effects Models (Laplace Approximation) for Parent/Caregiver-Reported Average Sleep Duration per Doubling of Serum Cotinine Concentration (log₂-Transformed) in the Marietta CARES Pediatric Cohort (Ages 7–9), 2008–2013 (n = 404).** | | | | |
| --- | --- | --- | --- | --- |
| **Age-adjusted** |  | Average sleeping hours~Log₂‑Transformed | | |
|  | n=404 | Estimate (95% CI) | p-value | in minutes |
| (Intercept) |  | 9.67 (8.68-10.7) | **<.0001** |  |
| Log₂‑Transformed (per doubling) |  | **-0.06 (-0.10, -0.03)** | **0.004** | **-3.81** |
| age (years) |  | -0.07 (-0.18, 0.05) | 0.27 | -3.94 |
|  |  |  |  |  |
| **Fully Adjusted** | n=404 | Estimate (95% CI) | p-value | in minutes |
| (Intercept) |  | 8.92 (7.68, 10.2) | **<.0001** |  |
| Log₂‑Transformed (per doubling) |  | -0.04 (-0.08, 0.001) | 0.06 | -2.37 |
| age (years) |  | -0.06 (-0.17, -0.06) | 0.34 | -3.44 |
| Sex |  | 0.04 (-0.17, 0.24) | 0.73 | 2.11 |
| Education |  | **0.05 (0.008, 0.10)** | **0.02** | **3.27** |
| BMI percentile |  | 0.001 (-0.004, 0.003) | 0.64 | -0.05 |
| episode |  | *non-estimable |  |  |
| Cotinine modeled on log₂ scale. PRs reflect the effect per doubling of serum cotinine concentration. | | | | |
| Parent education: Barratt Score. | | | | |
| Episodes: repeated episodes of difficulty breathing in the past 2 years. | | | | |
| The fully adjusted model additionally considers the following covariates: child sex, parent education, BMI percentile, and history of breathing difficulty in the past 2 years. | | | | |
| *Episode variable excluded due to non-estimable standard error in one imputed dataset. | | | | |

| **Supplemental Table 3.** **Prevalence Ratios (PR) and 95% Confidence Intervals (CI) for Parent/Caregiver-Reported Sleep Disturbances by Three Measures of SHS Exposure, Estimated Using PROC GLIMMIX (Fixed-Effects Model with Laplace Approximation), in the Marietta CARES Pediatric Cohort (Ages 7–9), 2008–2013 (n = 404).** | | | | | | |  |
| --- | --- | --- | --- | --- | --- | --- | --- |
| **Continuously measured Natural Log-Serum Cotinine** | |  |  |  |  |  |  |
| **Sleep Behavior** | Age-adjusted Association between Natural Log-Serum Cotinine and Sleep Behaviors | | | Fully Adjusted Model of Association between Natural Log-Serum Cotinine and Sleep Behaviors | | |  |
|  | **PR** | **95% CI** | **p-value** | **PR** | **95% CI** | **p-value** |  |
| Average Sleep (<8 hours vs. ≥8 hours) | **1.37** | **1.13-1.67** | **0.002** | **1.36** | **1.07-1.74** | **0.01** |  |
| Log₂‑Transformed (per doubling) * | **1.25** | **1.09-1.42** | **0.002** | **1.24** | **1.05-1.47** | **0.02** |  |
|  |  |  |  |  |  |  |  |
| Snoring (≥3 x/week vs. <3 x/week) | **1.13** | **1.02-1.25** | **0.02** | 1.10 | 0.98-1.24 | 0.11 |  |
| Log₂‑Transformed (per doubling) * | **1.09** | **1.01-1.16** | **0.02** | 1.07 | 0.99-1.16 | 0.11 |  |
|  |  |  |  |  |  |  |  |
| Night Awakenings (≥2 x/night vs. <2 x/night) | **1.28** | **1.01-1.63** | **0.04** | 1.24 | 0.93-1.65 | 0.56 |  |
| Log₂‑Transformed (per doubling) * | **1.19** | **1.01-1.40** | **0.04** | 1.16 | 0.95-1.41 | 0.14 |  |
|  |  |  |  |  |  |  |  |
| Number of sleep issues reported (≥1 vs. None) | **1.14** | **1.04-1.25** | **0.004** | **1.11** | **1.00-1.24** | **0.048** |  |
| Log₂‑Transformed (per doubling) * | **1.09** | **1.03-1.16** | **0.004** | **1.08** | **1.00-1.16** | **0.048** |  |
| **Dichotomized (≥0.05 vs. <0.05 ng/mL)** | | | | | | |  |
| **Sleep Behavior** | Age-adjusted Association between Parent/Caregiver Report of ≥1 Smoker in the Home and Sleep Behaviors | | | Fully Adjusted Model of Association between Natural Log-Serum Cotinine and Sleep Behaviors | | |  |
|  | **PR** | **95% CI** | **p-value** | **PR** | **95% CI** | **p-value** |  |
| Average Sleep (<8 hours vs. ≥8 hours) | **3.76** | **1.28-11.1** | **0.02** | 2.78 | 0.87-8.96 | 0.09 |  |
| Snoring (≥3 x/week vs. <3 x/week) | 1.49 | 0.96-2.32 | 0.07 | 1.30 | 0.80-2.11 | 0.28 |  |
| Night Awakenings (≥2 x/night vs. <2 x/night) | 2.53 | 0.67-9.60 | 0.17 | 1.89 | 0.41-8.69 | 0.41 |  |
| Number of sleep issues reported (≥1 vs. None) | **1.46** | **1.00-2.12** | **0.049** | 1.22 | 0.81-1.83 | 0.35 |  |
| **Parent/Caregiver-report of ≥1 Smoker in the Home** | | | | | | | |
| **Sleep Behavior** | Age-adjusted Association between Parent-Report of ≥1 Smoker in the Home and Sleep Behaviors | | | Fully Adjusted Model of Association between Natural Log-Serum Cotinine and Sleep Behaviors | | |  |
|  | **PR** | **95% CI** | **p-value** | **PR** | **95% CI** | **p-value** |  |
| Average Sleep (<8 hours vs. ≥8 hours) | **2.45** | **1.12-5.37** | **0.03** | 1.85 | 0.78-4.36 | 0.16 |  |
| Snoring (≥3 x/week vs. <3 x/week) | 1.39 | 0.94-2.04 | 0.10 | 1.18 | 0.77-1.80 | 0.44 |  |
| Night Awakenings (≥2 x/night vs. <2 x/night) | 2.47 | 0.96-6.31 | 0.06 | 1.89 | 0.66-5.36 | 0.24 |  |
| Number of sleep issues reported (≥1 vs. None) | 1.38 | 1.00-1.91 | 0.05 | 1.16 | 0.81-1.64 | 0.42 |  |
| *Cotinine additionally modeled on log₂ scale. PRs reflect the effect per doubling of serum cotinine concentration. | | | | | | |  |
| Age-adjusted model includes age (years). | | | | | | |  |
| The fully adjusted model additionally considers the following covariates: child sex, parent education, BMI percentile, and history of breathing difficulty in the past 2 years. | | | | | | |  |
